# Cognitive Symptoms of Post–COVID-19 Condition and Daily Functioning

**DOI:** 10.1001/jamanetworkopen.2023.56098

**Published:** 2024-02-14

**Authors:** Abhishek Jaywant, Faith M. Gunning, Lauren E. Oberlin, Mauricio Santillana, Katherine Ognyanova, James N. Druckman, Matthew A. Baum, David Lazer, Roy H. Perlis

**Affiliations:** 1 Department of Psychiatry, Weill Cornell Medicine, New York, New York; 2 Machine Intelligence Group for the Betterment of Health and the Environment, Northeastern University, Boston, Massachusetts; 3 Department of Epidemiology, Harvard T. H. Chan School of Public Health, Boston, Massachusetts; 4 Department of Communication, School of Communication and Information, Rutgers University, New Brunswick, New Jersey; 5 Department of Political Science, University of Rochester, Rochester, New York; 6 John F. Kennedy School of Government and Department of Government, Harvard University, Cambridge, Massachusetts; 7 Network Science Institute, Northeastern University, Boston, Massachusetts; 8 Department of Political Science, Northeastern University, Boston, Massachusetts; 9 Khoury College of Computer Sciences, Northeastern University, Boston, Massachusetts; 10 Institute for Quantitative Social Science, Harvard University, Cambridge, Massachusetts; 11 Center for Quantitative Health, Massachusetts General Hospital, Boston; 12 Department of Psychiatry, Harvard Medical School, Boston, Massachusetts

## Abstract

**Importance:**

The frequent occurrence of cognitive symptoms in post–COVID-19 condition has been described, but the nature of these symptoms and their demographic and functional factors are not well characterized in generalizable populations.

**Objective:**

To investigate the prevalence of self-reported cognitive symptoms in post–COVID-19 condition, in comparison with individuals with prior acute SARS-CoV-2 infection who did not develop post–COVID-19 condition, and their association with other individual features, including depressive symptoms and functional status.

**Design, Setting, and Participants:**

Two waves of a 50-state nonprobability population-based internet survey conducted between December 22, 2022, and May 5, 2023. Participants included survey respondents aged 18 years and older.

**Exposure:**

Post–COVID-19 condition, defined as self-report of symptoms attributed to COVID-19 beyond 2 months after the initial month of illness.

**Main Outcomes and Measures:**

Seven items from the Neuro-QoL cognition battery assessing the frequency of cognitive symptoms in the past week and patient Health Questionnaire-9.

**Results:**

The 14 767 individuals reporting test-confirmed COVID-19 illness at least 2 months before the survey had a mean (SD) age of 44.6 (16.3) years; 568 (3.8%) were Asian, 1484 (10.0%) were Black, 1408 (9.5%) were Hispanic, and 10 811 (73.2%) were White. A total of 10 037 respondents (68.0%) were women and 4730 (32.0%) were men. Of the 1683 individuals reporting post–COVID-19 condition, 955 (56.7%) reported at least 1 cognitive symptom experienced daily, compared with 3552 of 13 084 (27.1%) of those who did not report post–COVID-19 condition. More daily cognitive symptoms were associated with a greater likelihood of reporting at least moderate interference with functioning (unadjusted odds ratio [OR], 1.31 [95% CI, 1.25-1.36]; adjusted [AOR], 1.30 [95% CI, 1.25-1.36]), lesser likelihood of full-time employment (unadjusted OR, 0.95 [95% CI, 0.91-0.99]; AOR, 0.92 [95% CI, 0.88-0.96]) and greater severity of depressive symptoms (unadjusted coefficient, 1.40 [95% CI, 1.29-1.51]; adjusted coefficient 1.27 [95% CI, 1.17-1.38). After including depressive symptoms in regression models, associations were also found between cognitive symptoms and at least moderate interference with everyday functioning (AOR, 1.27 [95% CI, 1.21-1.33]) and between cognitive symptoms and lower odds of full-time employment (AOR, 0.92 [95% CI, 0.88-0.97]).

**Conclusions and Relevance:**

The findings of this survey study of US adults suggest that cognitive symptoms are common among individuals with post–COVID-19 condition and associated with greater self-reported functional impairment, lesser likelihood of full-time employment, and greater depressive symptom severity. Screening for and addressing cognitive symptoms is an important component of the public health response to post–COVID-19 condition.

## Key Points

**Question:**

How are post–COVID-19 condition self-reported cognitive symptoms associated with employment status, functional outcomes, and mood?

**Findings:**

In this survey study including 14 767 individuals with post–COVID-19 condition surveyed in late 2022 to early 2023, 57% reported experiencing cognitive symptoms daily, compared with 27% with prior SARS-CoV-2 infection who did not develop post–COVID-19 condition. In those with post–COVID-19 condition, cognitive symptoms were associated with greater levels of depressive symptoms, greater reported functional impairment, and lesser likelihood of full-time employment.

**Meaning:**

The findings of this study suggest that self-reported cognitive symptoms are prevalent in post–COVID-19 condition, often co-occur with depressive symptoms, and are associated with functional impairment.

## Teaser

This survey study examines self-reported cognitive symptoms of post–COVID-19 syndrome and the association of these symptoms with individuals' employment status, functional outcomes, and mood.

## Introduction

Persistence or late emergence of symptoms after acute infection with the SARS-CoV-2 virus, often referred to as long COVID^1^ or post-acute COVID-19 syndrome,^2^ is common. While estimates of the prevalence of post–COVID-19 condition vary widely depending on definition, methods, and sampling frame, studies suggest that at least 10% of individuals will experience at least some of these symptoms,^3,4,5^ including cognitive difficulties, fatigue, and mood changes.

Developing effective public health strategies for post–COVID-19 condition requires better understanding of the symptoms that compose this syndrome, their prevalence, and their association with functional outcomes.^2,6^ The recognition that the sequelae of COVID-19 represent a persistent public health crisis prompted a National Academy of Medicine effort to refine post–COVID-19 condition definitions and substantial investment from the US government to understand mechanisms and develop treatments for post–COVID-19 condition through the National Institutes of Health RECOVER initiative.^7^

In early work, cognitive deficits were reported to be common after acute COVID-19 illness.^8^ Subsequent work confirmed the persistence of deficits in attention, speed of information processing, executive functions, and memory.^9,10,11,12,13^ As recognition of post–COVID-19 condition as a syndrome emerged, cognition was initially investigated in small convenience samples of patients seeking care at single or multiple COVID-19–related specialty clinics,^12,14,15^ which may limit generalizability because of selection bias. Most larger studies have used *International Classification of Diseases* codes from electronic health records and health claims data,^3,13,16^ but individual signs or symptoms may not be captured reliably in encounter diagnostic codes.^3^ One exception is a large study in 2020 using a web-based platform^17^ that included 12 689 individuals in the UK with a suspected history of COVID-19 acute infection. That study identified cognitive deficits in multiple domains that were associated with the severity of COVID-19 acute infection but also observed persistence of cognitive weaknesses during the post–COVID-19 condition phase.

Beyond the individual cognitive symptoms of post–COVID-19 condition, their association with functioning has not been well established. Post–COVID-19 condition in general often compromises quality of life^18^ and has been associated with occupational difficulties^19^ and diminished everyday activities of daily living.^20^ A study of 3754 adults treated at a COVID-19 follow-up clinic from 2020 to March 2022 reported an association between self-reported cognitive symptoms and occupational and social function.^21^ Similarly, analysis of a cohort of patients seeking care at a COVID-19 clinic in 2020-2021 found that executive dysfunction demonstrated an association with work impairment and work absenteeism.^22^ Most recently, an association between self-reported cognitive impairment and unemployment in a survey-based design was reported.^23^ Still, the extent to which findings from early in the pandemic remain applicable to later infections is unknown, particularly as the risk of cognitive symptoms after COVID-19 may not have been stable through the course of the pandemic.^13,24,25^

To better understand the nature and correlates of cognitive symptoms in post–COVID-19 condition, we applied data from 2 waves of a 50-state US survey to characterize these symptoms among individuals who describe post–COVID-19 condition in comparison with those who report full recovery from SARS-CoV-2 infection. Data were collected from 14 767 individuals between December 22, 2022, and May 5, 2023, as part of a survey that contains questions on a broad array of topics, not solely COVID-19, to mitigate at least in part selection bias. We examined the association of these symptoms with individual respondent characteristics and then sought to evaluate the association between cognitive symptoms and mood and functional status.

## Methods

### Study Design

We incorporated data from 2 waves of the COVID States Project, an internet survey conducted by a consortium of academic sites (COVIDstates.org)^26^ between December 22, 2022, and January 7, 2023, and again April 5 to May 5, 2023, in all 50 US states and the District of Columbia. The survey enrolled individuals aged 18 years and older residing in the US, applying nonprobability sampling^27^ along with state-level representative quotas intended to balance the sample on age, gender, and race and ethnicity. Participants opted in via a commercial panel aggregator (Pure Spectrum) to participate in a general opinion survey; as the survey contained a broad range of questions, it was not promoted as a COVID survey per se. These individuals provided written online consent before accessing the survey, considered to be exempt by the Harvard University Institutional Review Board. All results are described in accordance with the American Association for Public Opinion Research (AAPOR) reporting guideline.^28^ As an opt-in survey, participation rates are not available.

### Measures

#### Cognitive Symptoms

Questions pertaining to cognitive symptoms were drawn from the Neuro-QoL v2.0 Cognitive Function item bank,^29^ a psychometrically validated patient-reported outcome measure^30^ that has been used in individuals recovering from COVID-19.^31^ Survey respondents were asked to rate on a 5-point Likert-type scale how often they experienced each of the following over the 7 previous days: slowed thinking, trouble concentrating, having to work hard to pay attention to avoid making mistakes, trouble getting started, trouble remembering (eg, taking medicine or buying something), difficulty multitasking, and trouble making decisions. By convention, lower scores on each item correspond to greater cognitive symptom frequency/burden as follows: 5, never; 4, rarely; 3, sometimes (2-3 times in the past 7 days); 2, often (once a day); and 1, very often (several times a day). For purposes of primary analysis, we counted a symptom as present if it was reported as occurring at least daily (ie, score of 2 or 1) and calculated the number of symptoms reported for each respondent. The Neuro-QOL does not ask whether symptoms are new or chronic, only whether they are present.

#### Mood

We used the 9-item Patient Health Questionnaire (PHQ-9) to screen for depressive symptom severity. The PHQ-9 has strong diagnostic accuracy in assessing depression.^32,33^ The PHQ-9 consists of 9 items that correspond to the 9 diagnostic criteria of the *Diagnostic and Statistical Manual of Mental Disorders, Fifth Edition* diagnosis of major depressive disorder. Respondents are asked to rate each item on a 0 to 3 Likert-type scale how frequently they experienced each symptom over the past 2 weeks (0, not at all; 3, nearly every day). Scores are summed to yield a total score.

#### Functional Outcomes

Survey respondents were asked about the extent to which post–COVID-19 condition symptoms interfered in daily life, with options including not at all, a little bit, moderately, quite a bit, and extremely; for analysis, this variable was dichotomized a priori to reflect moderate or greater, or less than moderately. Respondents were also asked about their current employment status, selecting from among working full-time, gig/contract work, homemaker, parttime work, retired, self-employed, student, or unemployed. For consistency with prior work^23^ and ease of interpretation, we used working full-time (yes vs all others) as a dichotomous variable in logistic regression analyses.

#### Sociodemographic Variables

Sociodemographic variables were collected by self-report. Race and ethnicity were drawn from US Census categories to confirm representativeness of the US population; we report these categories as specified in a recent guidance statement.^34^ Survey respondents were asked to identify race and ethnicity from a list including African American or Black, Asian American, Hispanic, Native American, Pacific Islander, White, or Other, and had the opportunity to provide a free-text self-description. For analytic purposes, because of small cell sizes, Native American, Pacific Islander, and Other were collapsed into a single category for analysis.

Post–COVID-19 condition and SARS-CoV-2 infection were defined based on participant self-report. Respondents were first asked whether they had received any positive COVID-19 test result and in what month they received this result. Those who answered affirmatively were further asked whether their symptoms had resolved. Among those indicating that symptoms had not resolved, they were asked to complete a checklist reflecting current acute and post–COVID-19 condition signs and symptoms.^25^ For consistency with prior work, we defined post–COVID-19 condition among individuals whose survey start date was more than 2 months after the month in which they initially identified a positive COVID-19 test and who continued to report symptoms at the time of the survey. This approximates the 2-month persistence criterion of the World Health Organization^35^ definition of post–COVID-19 condition, but not the 3-month interval from initial infection. Earlier work reported that alternate definitions relying on self-reported diagnosis rather than positive test results yielded similar results in terms of correlates and outcomes.^23^

### Statistical Analysis

All analyses were conducted using R, version 4.0 (R Foundation for Statistical Computing).^36^ Descriptive statistics were calculated using the mean (SD) for continuous measures and proportions for categorical variables. We graphically compared the mean score (representing frequency) on each Neuro-QoL item, and the total number of cognitive symptoms reported between those who met criteria for post–COVID-19 condition vs those who did not. Unadjusted odds ratios (ORs) and adjusted ORs (AORs) for presence of cognitive symptoms, for moderate or greater impact of symptoms on daily functioning, and for working full-time were estimated using multiple logistic regression, with adjusted models including sociodemographic features. Pearson correlations between test scores were determined. Linear regression was used to examine sociodemographic features for association with the overall number of symptoms reported. In all regression models, we considered the following features: age in years, gender, educational level (graduate, undergraduate, some college, high school graduate, and some high school or less), annual household income (<$25 000, $25 000-<$50 000, $50 000-<$100 000, and ≥$100 000), race and ethnicity, and rural, suburban, or urban setting. Survey weights were not applied as this represented a nonrandom subset of the larger sample, such that the aim was not to estimate US population rates but rather to derive a cohort of individuals with post–COVID-19 condition based on this larger sample. For participants who responded to more than 1 survey wave, the most recent survey was included; in prior work,^37^ alternative approaches (ie, using index wave or a random wave, or including multiple within-subject measures and accounting for nested observations) yielded essentially identical results. In light of very low rates of missing data (Table 1), we did not apply multiple imputation. The threshold for statistical significance was considered to be 2-sided *P* < .05.

**Table 1.** Characteristics of Individuals Who Did or Did Not Report Persistence of Post–COVID-19 Condition Symptoms for at Least 2 Months

Characteristic	No. (%)			*P* value
	Recovered (n = 13 084)	Post–COVID-19 condition (n = 1683)	Total (N = 14 767)	
Respondent age, mean (SD), y	44.3 (16.5)	46.9 (14.9)	44.6 (16.3)	<.001
Gender				
Female	8704 (66.5)	1333 (79.2)	10 037 (68.0)	<.001
Male	4380 (33.5)	350 (20.8)	4730 (32.0)	
Race and ethnicity				
African American or Black	1355 (10.4)	129 (7.7)	1484 (10.0)	<.001
Asian American	540 (4.1)	28 (1.7)	568 (3.8)	
Hispanic	1253 (9.6)	155 (9.2)	1408 (9.5)	
Native American	126 (1.0)	18 (1.1)	144 (1.0)	
Other^a^	160 (1.2)	32 (1.9)	192 (1.3)	
Pacific Islander	132 (1.0)	28 (1.7)	160 (1.1)	
White	9518 (72.7)	1293 (76.8)	10 811 (73.2)	
Education				
Some high school	338 (2.6)	58 (3.4)	396 (2.7)	<.001
High school degree	2606 (19.9)	358 (21.3)	2964 (20.1)	
Some college	3155 (24.1)	525 (31.2)	3680 (24.9)	
College degree	5214 (39.9)	557 (33.1)	5771 (39.1)	
Graduate degree	1771 (13.5)	185 (11.0)	1956 (13.2)	
Employment				
Full-time	6275 (48.0)	669 (39.8)	6944 (47.0)	<.001
Gig/contract	54 (0.4)	10 (0.6)	64 (0.4)	
Homemaker	830 (6.3)	127 (7.5)	957 (6.5)	
Parttime	1362 (10.4)	173 (10.3)	1535 (10.4)	
Retired	2143 (16.4)	319 (19.0)	2462 (16.7)	
Self-employed	747 (5.7)	128 (7.6)	875 (5.9)	
Student	590 (4.5)	46 (2.7)	636 (4.3)	
Unemployed	1083 (8.3)	211 (12.5)	1294 (8.8)	
Income, $				
Missing	5	1	6	<.001
<25 000	2135 (16.3)	354 (21.0)	2489 (16.9)	
25 000 to < 50 000	3128 (23.9)	485 (28.8)	3613 (24.5)	
50 000 to <100 000	4588 (35.1)	572 (34.0)	5160 (35.0)	
≥100 000	3228 (24.7)	271 (16.1)	3499 (23.7)	
Urbanicity				
Rural	2191 (16.7)	397 (23.6)	2588 (17.5)	<.001
Suburban	7650 (58.5)	959 (57.0)	8609 (58.3)	
Urban	3243 (24.8)	327 (19.4)	3570 (24.2)	
PHQ-9 total score				
Not administered	246	29	275	<.001
Mean (SD)	6.2 (6.3)	9.5 (7.0)	6.6 (6.5)	
Daily cognitive symptoms, mean (SD)	0.8 (1.7)	2.3 (2.6)	1.0 (1.9)	<.001
At least 1 cognitive symptom	3552 (27.1)	955 (56.7)	4507 (30.5)	<.001

Abbreviation: PHQ-9, 9-item Patient Health Questionnaire.

^a^ Individuals who did not select one of the other categories listed for race and ethnicity could instead indicate Other and respond using free text.

## Results

Among all 14 767 individuals reporting test-confirmed SARS-CoV-2 infection at least 2 months before the survey (Table 1), mean (SD) age was 44.6 (16.3) years; 568 (3.8%) were Asian, 1484 (10.0%) were Black, 1408 (9.5%) were Hispanic, and 10 811 (73.2%) were White. A total of 10 037 respondents 68.0%) were women and 4730 (32.0%) were men. A total of 1683 individuals (11.4%) of the full sample met our definition of post–COVID-19 condition. Of the 1683 individuals reporting post–COVID-19 condition, 955 (56.7%) reported at least 1 cognitive symptom experienced daily, compared with 3552 of 13 084 (27.1%) of those who did not report post–COVID-19 condition.

**Figure 1.**
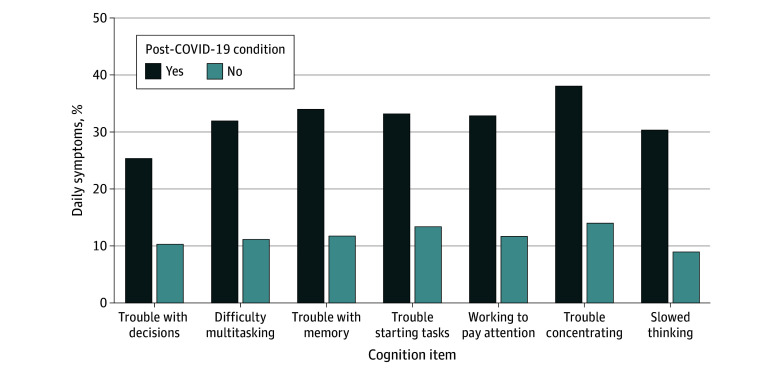
Proportion of Individuals Reporting Individual Cognitive Symptoms Occurring at Least Daily

All individual cognitive symptoms were more often reported as occurring at least daily among respondents with post–COVID-19 condition (Figure 1), ranging from trouble with decision-making (25.3%) to trouble concentrating (38.1%), and occurred more frequently overall (eFigure 1 in Supplement 1). Those with post–COVID-19 condition reported, on average, significantly more cognitive symptoms experienced at least daily than those without post–COVID-19 condition (eFigure 2 in Supplement 1).

**Figure 2.**
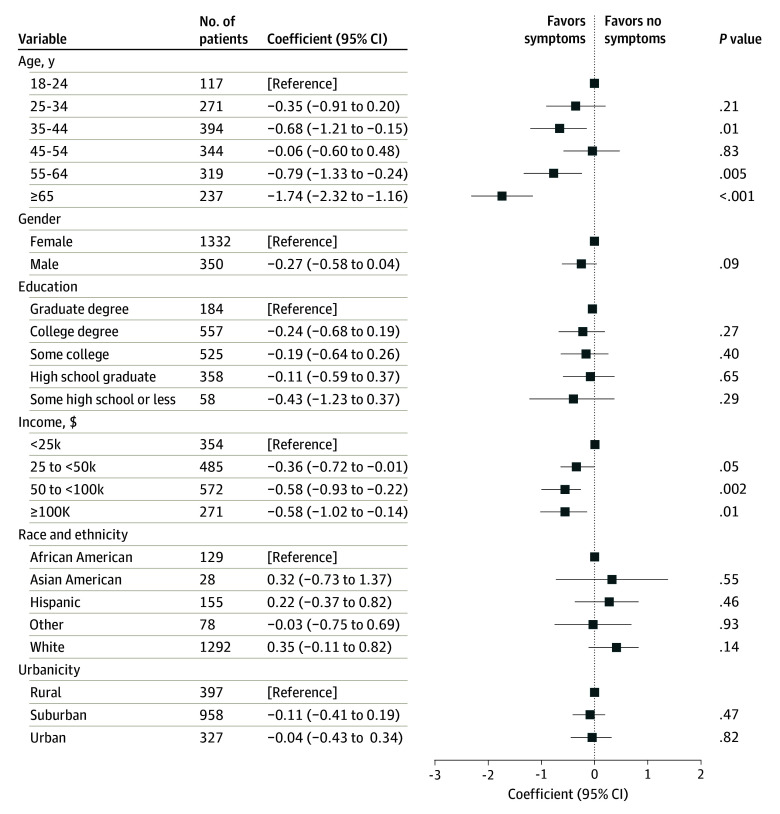
Linear Regression Model of Number of Cognitive Symptoms Associated With Sociodemographic Features Individuals who did not select one of the other categories listed for race and ethnicity could instead indicate Other and respond using free text. African American includes African American or Black. OR indicates odds ratio.

Among individuals with post–COVID-19 condition, we next used linear regression models to compare individual sociodemographic features with the number of cognitive symptoms reported at least daily. Features associated with a greater number of daily symptoms included younger age and lower income (Figure 2), although age reflected a nonlinear pattern, with greatest symptoms in the 18- to 24-year and 45- to 54-year groups, and least among the 65 years and older group. Characteristics of individuals with or without at least 1 symptom are summarized in Table 2 and the eTable in Supplement 1.

**Table 2.** Individuals With Post–COVID-19 Condition Who Did or Did Not Report Any Cognitive Symptoms Occurring at Least Daily

Characteristic	No. (%)			*P* value
	No cognitive symptoms (n = 728)	Cognitive symptoms (n = 955)	Total (n = 1683)	
Respondent age, mean (SD), y	49.8 (15.2)	44.6 (14.2)	46.9 (14.9)	<.001
Range	18.0-87.0	18.0-86.0	18.0-87.0	
Gender				
Female	549 (75.4)	784 (82.1)	1333 (79.2)	<.001
Male	179 (24.6)	171 (17.9)	350 (20.8)	
Race and ethnicity				
African American or Black	60 (8.2)	69 (7.2)	129 (7.7)	.87
Asian American	12 (1.6)	16 (1.7)	28 (1.7)	
Hispanic	66 (9.1)	89 (9.3)	155 (9.2)	
Other^a^	37 (5.1)	41 (4.3)	78 (4.6)	
White	553 (76.0)	740 (77.5)	1293 (76.8)	
Education				
Graduate degree	80 (11.0)	105 (11.0)	185 (11.0)	.56
College degree	256 (35.2)	301 (31.5)	557 (33.1)	
Some college	220 (30.2)	305 (31.9)	525 (31.2)	
High school graduate	150 (20.6)	208 (21.8)	358 (21.3)	
Some high school or less	22 (3.0)	36 (3.8)	58 (3.4)	
Income, $				
Missing	0	1	1	.08
<25 000	135 (18.5)	219 (23.0)	354 (21.0)	
25 000 to < 50 000	205 (28.2)	280 (29.4)	485 (28.8)	
50 000 to <100 000	262 (36.0)	310 (32.5)	572 (34.0)	
≥100 000	126 (17.3)	145 (15.2)	271 (16.1)	
Urbanicity				
Rural	164 (22.5)	233 (24.4)	397 (23.6)	.61
Suburban	424 (58.2)	535 (56.0)	959 (57.0)	
Urban	140 (19.2)	187 (19.6)	327 (19.4)	
Impairment				
Not at all	133 (18.3)	62 (6.5)	195 (11.6)	<.001
A little bit	407 (55.9)	413 (43.2)	820 (48.7)	
Moderately	127 (17.4)	277 (29.0)	404 (24.0)	
Quite a bit	44 (6.0)	143 (15.0)	187 (11.1)	
Extremely	17 (2.3)	60 (6.3)	77 (4.6)	
Employment				
Full-time	296 (40.7)	373 (39.1)	669 (39.8)	<.001
Gig/contract	1 (0.1)	9 (0.9)	10 (0.6)	
Homemaker	48 (6.6)	79 (8.3)	127 (7.5)	
Parttime	71 (9.8)	102 (10.7)	173 (10.3)	
Retired	183 (25.1)	136 (14.2)	319 (19.0)	
Self-employed	45 (6.2)	83 (8.7)	128 (7.6)	
Student	15 (2.1)	31 (3.2)	46 (2.7)	
Unemployed	69 (9.5)	142 (14.9)	211 (12.5)	
PHQ-9 total score				
Not administered	10	19	29	<.001
Mean (SD)	6.1 (5.4)	12.2 (6.9)	9.5 (7.0)	

Abbreviation: PHQ-9, 9-item Patient Health Questionnaire.

^a^ Other includes Native American, Pacific Islander, or individuals who identified “Other” and responded using free text.

**Figure 3.**
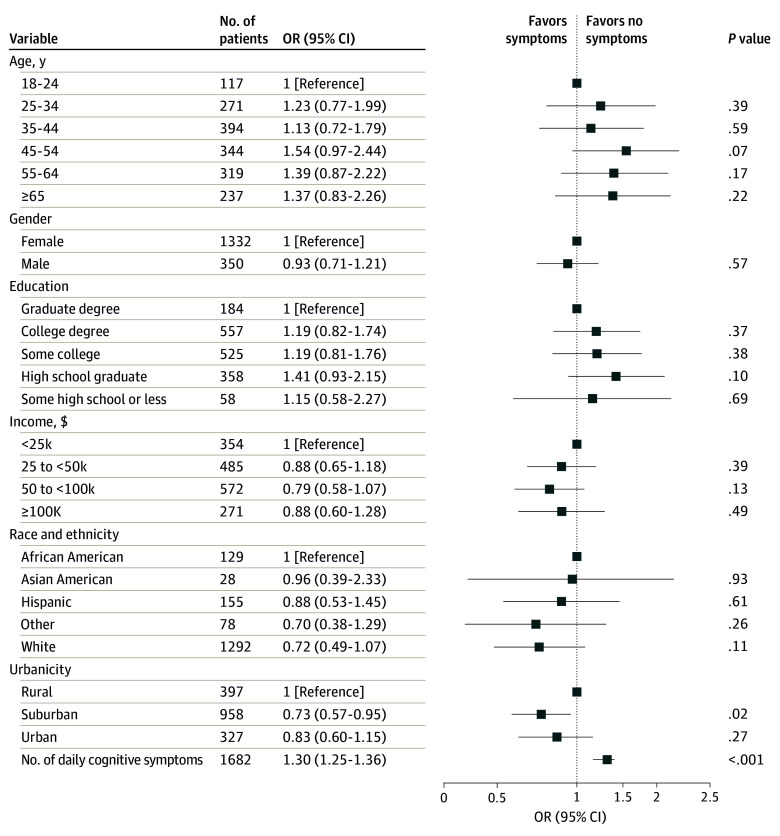
Logistic Regression Model of Full-Time Employment Examining Number of Cognitive Symptoms Reported at Least Daily and Sociodemographic Features Individuals who did not select one of the other categories listed for race and ethnicity could instead indicate Other and respond using free text. African American includes African American or Black. OR indicates odds ratio.

We further examined the number of cognitive symptoms reported daily among individuals with post–COVID-19 condition and mood and daily functioning. In regression models, the number of cognitive symptoms reported at least daily was associated with greater severity of depressive symptoms on the PHQ-9 (unadjusted coefficient, 1.40 [95% CI, 1.29-1.51]; adjusted coefficient, 1.27 [95% CI, 1.17-1.38]) (eFigure 3 in Supplement 1). eFigure 4 in Supplement 1 shows a heatmap of correlations between Neuro-QoL item scores and PHQ-9 item scores. More daily cognitive symptoms were associated with a greater likelihood of reporting at least moderate interference with functioning (unadjusted OR, 1.31 [95% CI, 1.26-1.36]; AOR, 1.30 [95% CI, 1.25-1.36]) (Figure 3) as well as lesser likelihood of full-time employment (unadjusted OR, 0.95 [95% CI, 0.91-0.99]; AOR, 0.92 [95% CI, 0.88-0.96]) (eFigure 5 in Supplement 1). After including depressive symptoms in regression models, significant findings noted in crude models between cognitive symptoms and at least moderate interference with functioning were also significant in adjusted models (AOR, 1.27 [95% CI, 1.21-1.33]) and between cognitive symptoms and employment status (AOR, 0.92 [95% CI, 0.88-0.97]) (eFigure 6 and eFigure 7 in Supplement 1).

## Discussion

In this survey-based cohort of 14 767 individuals with past or current COVID-19 symptoms, 955 of 1683 (56.7%) of those with post–COVID-19 condition described experiencing at least 1 cognitive symptom on a daily basis, substantially more than those without post–COVID-19 condition (27.1%). Prevalence of these symptoms was markedly different between sociodemographic subgroups, with greater cognitive symptoms reported among younger individuals, women, and those with lower levels of income.

Our finding that more than 50% of individuals with post–COVID-19 condition experience at least 1 cognitive symptom on a daily basis is consistent with 46% of individuals with post–COVID-19 condition in a survey of 2359 US adults that asked more generally about cognition.^25^ Our results extend a growing body of research on the cognitive sequelae of COVID-19, clarifying both the nature of such symptoms and their correlates in a nationally representative cohort reflective of late-pandemic post–COVID-19 condition prevalence.

Our finding of greater daily cognitive symptoms in post–COVID-19 condition in women vs men aligns with prior work that has reported female sex as a risk factor for post–COVID-19 condition.^38,39^ That younger age is associated with increased daily cognitive symptoms may reflect increased salience of symptoms or change from pre-COVID-19 baseline, in younger individuals relative to older individuals who may already be experiencing age-associated cognitive decline. The apparent nonlinear association herein merits further study, as the age 45- to 54-year group also exhibited substantial rates of such symptoms similar to those of the 18- to 24-year group. The association between cognitive symptoms and lower income suggests that economic stress and its correlates may confer vulnerability to cognitive sequelae in post–COVID-19 condition.

We further found that individuals with a greater number of cognitive symptoms also have a significantly greater burden of depressive symptoms, on average. As both sets of symptoms may reflect brain involvement in COVID-19 sequelae, this association is not unexpected. However, to date, the role of depressive symptoms in post–COVID-19 condition has received relatively little study.^40,41^ In part, this may represent the complexity of examining causation. Individuals with post–COVID-19 condition, particularly in the pandemic, may have initially been diagnosed with depression or anxiety, before post–COVID-19 condition as a syndrome was recognized. Our observation of the overlap between cognition and mood in post–COVID-19 condition does not in any way suggest any causal relationship, as the association may be bidirectional and/or may reflect a shared underlying mechanism.

Cognitive symptoms were also associated with greater likelihood of describing moderate or greater difficulties of post–COVID-19 condition symptoms in daily life and reduced likelihood of full-time employment. These findings extend the prior report^23^ and early work conducted in the initial phases of the pandemic that suggested associations between cognitive dysfunction, working capacity, and daily functioning.^19,42^ Neither association was meaningfully changed by incorporating depressive symptoms in regression models, suggesting that cognitive symptoms are not simply a proxy for or manifestation of depression. This finding is in line with that of Walker et al,^21^ who reported that cognitive dysfunction, but not depression, was associated with the ability to work after COVID-19. More broadly, our results further underscore the potential economic impact of post–COVID-19 condition.^43^

Taken together, these results suggest the importance of considering cognitive symptoms in the evaluation and management of post–COVID-19 condition, as they are associated with differences in functioning (as measured herein by self-report of daily impact, as well as employment status) and quality of life (as measured herein by depressive symptoms). They also underscore the need to better understand the biological mechanism underlying these factors to develop effective treatments. Preliminary work indicates, for example, that cognitive dysfunction may be associated with altered gray matter volume, white matter integrity, and functional connectivity in cortical, subcortical, and cerebellar regions.^44,45^

### Limitations

Our study has multiple limitations. Most important, since data collection uses a nonprobability design in which participants opt in, we cannot reliably calculate response rate or estimate nonresponse bias. Prior validation studies from this survey suggest concordance with probability-sampled polls and administrative data.^46,47^ We also cannot avoid reliance on self-report, including estimates of illness duration. As such, our results complement but do not replace other designs, allowing prospective longitudinal assessment, including objective cognitive testing. The cross-sectional nature of the study also precludes comparison with the prevalence of pre–COVID-19 symptoms. As a broad study not linked to detailed medical history, we cannot examine the association with other comorbidities or treatments that may affect cognition independent of COVID-19 or that may moderate the effects of COVID-19. We also elected not to examine interval from infection or severity of initial infection, previously associated with cognitive symptoms,^48^ as recall for each of these may be poor. In addition, while we examined associations between cognitive symptoms and depressive symptoms, we note that some items on the PHQ-9 (for example, fatigue and motor slowness) may also capture COVID-19 somatic symptoms rather than features of major depression per se. Prior investigation suggested the validity of the PHQ-9 even in individuals with a range of neurologic disorders.^49^

## Conclusions

The results of this survey study suggest that cognitive symptoms represent common features of post–COVID-19 condition, encompassing impairments often characterized by the widely used lay term brain fog. In light of their marked association with poorer functioning and quality of life, these symptoms represent important targets for assessment and identifying scalable interventions to remediate cognitive dysfunction in post–COVID-19 condition.

## Notes

Supplement 1. **eFigure 1.** Mean Score by Item, Long COVID Present Versus Absent **eFigure 2.** Count of Symptoms Reported Daily Among Those With a Prior History of COVID-19 **eTable.** Individuals With Long COVID Who Did, or Did Not, Report Any Cognitive Symptoms Occurring at Least Daily – Row Rather Than Column Totals **eFigure 3.** Linear Regression Model of PHQ-9 **eFigure 4.** Correlations Between Individual Cognitive and PHQ-9 Items **eFigure 5.** Logistic Regression Model of Full-time Employment, Examining Number of Cognitive Symptoms Endorsed at Least Daily **eFigure 6.** Logistic Regression Model of Moderate or Greater Functional Impairment, Examining Number of Cognitive Symptoms Endorsed at Least Daily, Including PHQ9 Total Score **eFigure 7.** Logistic Regression Model of Full-time Employment, Examining Number of Cognitive Symptoms Endorsed at Least Daily, Including PHQ9 Total Score

Supplement 2.
Data Sharing Statement

## References

[1] NICE. COVID-19 rapid guideline: managing the long-term effects of COVID-19. November 11, 2021. Accessed April 30, 2021. https://www.nice.org.uk/guidance/ng188

[2] Nalbandian A, Sehgal K, Gupta A, . Post-acute COVID-19 syndrome. Nat Med. 2021;27(4):601-615. doi:10.1038/s41591-021-01283-z 33753937 PMC8893149

[3] Castro VM, Rosand J, Giacino JT, McCoy TH, Perlis RH. Case-control study of neuropsychiatric symptoms in electronic health records following COVID-19 hospitalization in 2 academic health systems. Mol Psychiatry. 2022;27(9):3898-3903. doi:10.1038/s41380-022-01646-z 35705635 PMC9199464

[4] Sudre CH, Murray B, Varsavsky T, . Attributes and predictors of long COVID. Nat Med. 2021;27(4):626-631. doi:10.1038/s41591-021-01292-y 33692530 PMC7611399

[5] Al-Aly Z, Xie Y, Bowe B. High-dimensional characterization of post-acute sequelae of COVID-19. Nature. 2021;594(7862):259-264. doi:10.1038/s41586-021-03553-9 33887749

[6] Honigsbaum M, Krishnan L. Taking pandemic sequelae seriously: from the Russian influenza to COVID-19 long-haulers. Lancet. 2020;396(10260):1389-1391. doi:10.1016/S0140-6736(20)32134-6 33058777 PMC7550169

[7] Thaweethai T, Jolley SE, Karlson EW, ; RECOVER Consortium. Development of a definition of postacute sequelae of SARS-CoV-2 Infection. JAMA. 2023;329(22):1934-1946. doi:10.1001/jama.2023.8823 37278994 PMC10214179

[8] Jaywant A, Vanderlind WM, Alexopoulos GS, Fridman CB, Perlis RH, Gunning FM. Frequency and profile of objective cognitive deficits in hospitalized patients recovering from COVID-19. Neuropsychopharmacology. 2021;46(13):2235-2240. doi:10.1038/s41386-021-00978-8 33589778 PMC7884062

[9] Almeria M, Cejudo JC, Sotoca J, Deus J, Krupinski J. Cognitive profile following COVID-19 infection: clinical predictors leading to neuropsychological impairment. Brain Behav Immun Health. 2020;9(October):100163. doi:10.1016/j.bbih.2020.100163 33111132 PMC7581383

[10] Becker JH, Lin JJ, Doernberg M, . Assessment of cognitive function in patients after COVID-19 infection. JAMA Netw Open. 2021;4(10):e2130645. doi:10.1001/jamanetworkopen.2021.30645 34677597 PMC8536953

[11] Ceban F, Ling S, Lui LMW, . Fatigue and cognitive impairment in post-COVID-19 syndrome: a systematic review and meta-analysis. Brain Behav Immun. 2022;101:93-135. doi:10.1016/j.bbi.2021.12.020 34973396 PMC8715665

[12] Vannorsdall TD, Brigham E, Fawzy A, . Cognitive dysfunction, psychiatric distress, and functional decline after COVID-19. J Acad Consult Liaison Psychiatry. 2022;63(2):133-143. doi:10.1016/j.jaclp.2021.10.006 34793996 PMC8591857

[13] Taquet M, Sillett R, Zhu L, . Neurological and psychiatric risk trajectories after SARS-CoV-2 infection: an analysis of 2-year retrospective cohort studies including 1 284 437 patients. Lancet Psychiatry. 2022;9(10):815-827. doi:10.1016/S2215-0366(22)00260-7 35987197 PMC9385200

[14] Sneller MC, Liang CJ, Marques AR, . A longitudinal study of COVID-19 sequelae and immunity: baseline findings. Ann Intern Med. 2022;175(7):969-979. doi:10.7326/M21-4905 35605238 PMC9128805

[15] Stallmach A, Kesselmeier M, Bauer M, . Comparison of fatigue, cognitive dysfunction and psychological disorders in post-COVID patients and patients after sepsis: is there a specific constellation? Infection. 2022;50(3):661-669. doi:10.1007/s15010-021-01733-3 34997542 PMC8741139

[16] Taquet M, Luciano S, Geddes JR, Harrison PJ. Bidirectional associations between COVID-19 and psychiatric disorder: retrospective cohort studies of 62 354 COVID-19 cases in the USA. Lancet Psychiatry. 2021;8(2):130-140. doi:10.1016/S2215-0366(20)30462-4 33181098 PMC7820108

[17] Hampshire A, Trender W, Chamberlain SR, . Cognitive deficits in people who have recovered from COVID-19. EClinicalMedicine. 2021;39:101044. doi:10.1016/j.eclinm.2021.101044 34316551 PMC8298139

[18] Malik P, Patel K, Pinto C, . Post-acute COVID-19 syndrome (PCS) and health-related quality of life (HRQoL)—a systematic review and meta-analysis. J Med Virol. 2022;94(1):253-262. doi:10.1002/jmv.27309 34463956 PMC8662132

[19] Davis HE, Assaf GS, McCorkell L, . Characterizing long COVID in an international cohort: 7 months of symptoms and their impact. EClinicalMedicine. 2021;38:101019. doi:10.1016/j.eclinm.2021.101019 34308300 PMC8280690

[20] Callan C, Ladds E, Husain L, Pattinson K, Greenhalgh T. “I can’t cope with multiple inputs”: a qualitative study of the lived experience of “brain fog” after COVID-19. BMJ Open. 2022;12(2):e056366. doi:10.1136/bmjopen-2021-056366 35149572 PMC8844964

[21] Walker S, Goodfellow H, Pookarnjanamorakot P, . Impact of fatigue as the primary determinant of functional limitations among patients with post-COVID-19 syndrome: a cross-sectional observational study. BMJ Open. 2023;13(6):e069217. doi:10.1136/bmjopen-2022-069217 37286327 PMC10335413

[22] Miskowiak KW, Pedersen JK, Gunnarsson DV, . Cognitive impairments among patients in a long-COVID clinic: prevalence, pattern and relation to illness severity, work function and quality of life. J Affect Disord. 2023;324:162-169. doi:10.1016/j.jad.2022.12.122 36586593 PMC9795797

[23] Perlis RH, Lunz Trujillo K, Safarpour A, . Association of post-COVID-19 condition symptoms and employment status. JAMA Netw Open. 2023;6(2):e2256152. doi:10.1001/jamanetworkopen.2022.56152 36790806 PMC9932847

[24] Romero-Rodríguez E, Pérula-de Torres LÁ, Castro-Jiménez R, . Hospital admission and vaccination as predictive factors of long COVID-19 symptoms. Front Med (Lausanne). 2022;9:1016013. doi:10.3389/fmed.2022.1016013 36438042 PMC9691755

[25] Perlis RH, Santillana M, Ognyanova K, . Prevalence and correlates of long COVID symptoms among US adults. JAMA Netw Open. 2022;5(10):e2238804. doi:10.1001/jamanetworkopen.2022.38804 36301542 PMC9614581

[26] The COVID States Project. Accessed January 4, 2024. https://www.covidstates.org/

[27] Kennedy C, Caumont R. What we learned about online nonprobability polls. Pew Research Center. May 2, 2016. Accessed November 27, 2021. https://www.pewresearch.org/fact-tank/2016/05/02/q-a-online-nonprobability-polls/

[28] Survey Disclosure Checklist - AAPOR. Accessed January 25, 2022. https://aapor.org/wp-content/uploads/2022/11/TI-Attachment-E.pdf

[29] Gershon RC, Lai JS, Bode R, . Neuro-QOL: quality of life item banks for adults with neurological disorders: item development and calibrations based upon clinical and general population testing. Qual Life Res. 2012;21(3):475-486. doi:10.1007/s11136-011-9958-8 21874314 PMC3889669

[30] Cella D, Lai JS, Nowinski CJ, . Neuro-QOL: brief measures of health-related quality of life for clinical research in neurology. Neurology. 2012;78(23):1860-1867. doi:10.1212/WNL.0b013e318258f744 22573626 PMC3369516

[31] Tabacof L, Tosto-Mancuso J, Wood J, . Post-acute COVID-19 syndrome negatively impacts physical function, cognitive function, health-related quality of life, and participation. Am J Phys Med Rehabil. 2022;101(1):48-52. doi:10.1097/PHM.0000000000001910 34686631 PMC8667685

[32] Kroenke K, Spitzer RL, Williams JBW. The PHQ-9: validity of a brief depression severity measure. J Gen Intern Med. 2001;16(9):606-613. doi:10.1046/j.1525-1497.2001.016009606.x 11556941 PMC1495268

[33] Levis B, Benedetti A, Thombs BD; DEPRESsion Screening Data (DEPRESSD) Collaboration. Accuracy of Patient Health Questionnaire-9 (PHQ-9) for screening to detect major depression: individual participant data meta-analysis. BMJ. 2019;365:l1476. doi:10.1136/bmj.l1476 30967483 PMC6454318

[34] Flanagin A, Frey T, Christiansen SL; AMA Manual of Style Committee. Updated guidance on the reporting of race and ethnicity in medical and science journals. JAMA. 2021;326(7):621-627. doi:10.1001/jama.2021.13304 34402850

[35] World Health Organization. A clinical case definition of post COVID-19 condition by a Delphi consensus. October 6, 2021. Accessed June 21, 2022. https://www.who.int/publications-detail-redirect/WHO-2019-nCoV-Post_COVID-19_condition-Clinical_case_definition-2021.1

[36] R Core Team. R: A language and environment for statistical computing. 2019. Accessed August 11, 2023. http://www.R-project.org

[37] Perlis RH, Lunz Trujillo K, Safarpour A, . Community mobility and depressive symptoms during the COVID-19 pandemic in the United States. JAMA Netw Open. 2023;6(9):e2334945. doi:10.1001/jamanetworkopen.2023.34945 37755830 PMC10534266

[38] Pihlaja RE, Kauhanen LS, Ollila HS, . Associations of subjective and objective cognitive functioning after COVID-19: a six-month follow-up of ICU, ward, and home-isolated patients. Brain Behav Immun Health. 2023;27:100587. doi:10.1016/j.bbih.2023.100587 36624888 PMC9812472

[39] Cavaco S, Sousa G, Gonçalves A, . Predictors of cognitive dysfunction one-year post COVID-19. Neuropsychology. 2023;37(5):557-567. doi:10.1037/neu0000876 36603126

[40] Perlis RH, Ognyanova K, Santillana M, . Association of acute symptoms of COVID-19 and symptoms of depression in adults. JAMA Netw Open. 2021;4(3):e213223. doi:10.1001/jamanetworkopen.2021.3223 33710287 PMC7955267

[41] Perlis RH, Santillana M, Ognyanova K, . Factors associated with self-reported symptoms of depression among adults with and without a previous COVID-19 diagnosis. JAMA Netw Open. 2021;4(6):e2116612. doi:10.1001/jamanetworkopen.2021.16612 34115130 PMC8196339

[42] Peter RS, Nieters A, Kräusslich HG, ; EPILOC Phase 1 Study Group. Post-acute sequelae of covid-19 six to 12 months after infection: population based study. BMJ. 2022;379:e071050. doi:10.1136/bmj-2022-071050 36229057 PMC9557001

[43] Is “long Covid” worsening the labor shortage? Brookings. January 11, 2022. Accessed July 13, 2023. https://www.brookings.edu/articles/is-long-covid-worsening-the-labor-shortage/

[44] Douaud G, Lee S, Alfaro-Almagro F, . SARS-CoV-2 is associated with changes in brain structure in UK Biobank. Nature. 2022;604(7907):697-707. doi:10.1038/s41586-022-04569-5 35255491 PMC9046077

[45] Díez-Cirarda M, Yus M, Gómez-Ruiz N, . Multimodal neuroimaging in post-COVID syndrome and correlation with cognition. Brain. 2023;146(5):2142-2152. doi:10.1093/brain/awac384 36288544 PMC9620345

[46] Radford J, Green J, Quintana A, . Evaluating the generalizability of the COVID States survey—a large-scale, non-probability survey. OSF Preprints. Preprint posted online March 7, 2022. doi:10.31219/osf.io/cwkg7

[47] Perlis RH, Simonson MD, Green J, . Prevalence of firearm ownership among individuals with major depressive symptoms. JAMA Netw Open. 2022;5(3):e223245. doi:10.1001/jamanetworkopen.2022.3245 35311961 PMC8938748

[48] Liu YH, Chen Y, Wang QH, . One-year trajectory of cognitive changes in older survivors of COVID-19 in Wuhan, China: a longitudinal cohort study. JAMA Neurol. 2022;79(5):509-517. doi:10.1001/jamaneurol.2022.0461 35258587 PMC8905512

[49] Katzan IL, Lapin B, Griffith S, . Somatic symptoms have negligible impact on Patient Health Questionnaire-9 depression scale scores in neurological patients. Eur J Neurol. 2021;28(6):1812-1819. doi:10.1111/ene.14822 33715277

