# Supplementary material for: Cognitive Symptoms of Post–COVID-19 Condition and Daily Functioning
Source: JAMA Netw Open. 2024 Feb 14;7(2):e2356098. doi: 10.1001/jamanetworkopen.2023.56098 (PMC10867690; doi:10.1001/jamanetworkopen.2023.56098)
Supplement: Supplement 1. — eFigure 1. Mean Score by Item, Long COVID Present Versus Absent eFigure 2. Count of Symptoms Reported Daily Among Those With a Prior History of COVID-19 eTable. Individuals With Long COVID Who Did, or Did Not, Report Any Cognitive Symptoms Occurring at Least Daily – Row Rather Than Column Totals eFigure 3. Linear Regression Model of PHQ-9 eFigure 4. Correlations Between Individual Cognitive and PHQ-9 Items eFigure 5. Logistic Regression Model of Full-time Employment, Examining Number of Cognitive Symptoms Endorsed at Least Daily eFigure 6. Logistic Regression Model of Moderate or Greater Functional Impairment, Examining Number of Cognitive Symptoms Endorsed at Least Daily, Including PHQ9 Total Score eFigure 7. Logistic Regression Model of Full-time Employment, Examining Number of Cognitive Symptoms Endorsed at Least Daily, Including PHQ9 Total Score [file jamanetwopen-e2356098-s001.pdf]

Supplementary Online Content

Jaywant A, Gunning FM, Oberlin LE, et al. Cognitive symptoms of post–COVID-19 condition and daily functioning. *JAMA Netw Open*. 2024;7(2):e2356098. doi:10.1001/jamanetworkopen.2023.56098

eFigure 1. Mean Score by Item, Long COVID Present Versus Absent

eFigure 2. Count of Symptoms Reported Daily Among Those With a Prior History of COVID-19

eTable. Individuals With Long COVID Who Did, or Did Not, Report Any Cognitive Symptoms Occurring at Least Daily – Row Rather Than Column Totals

eFigure 3. Linear Regression Model of PHQ-9

eFigure 4. Correlations Between Individual Cognitive and PHQ-9 Items

eFigure 5. Logistic Regression Model of Full-time Employment, Examining Number of Cognitive Symptoms Endorsed at Least Daily

eFigure 6. Logistic Regression Model of Moderate or Greater Functional Impairment, Examining Number of Cognitive Symptoms Endorsed at Least Daily, Including PHQ9 Total Score

eFigure 7. Logistic Regression Model of Full-time Employment, Examining Number of Cognitive Symptoms Endorsed at Least Daily, Including PHQ9 Total Score

This supplementary material has been provided by the authors to give readers additional information about their work.

© 2024 Jaywant A et al. *JAMA Network Open.*

eFigure 1. Mean score by item, long COVID present versus absent.

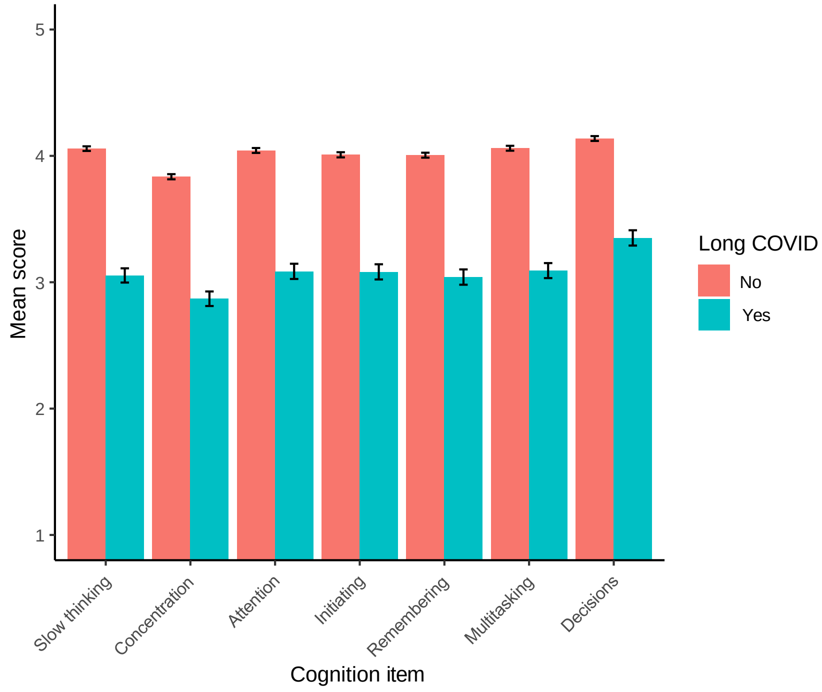

Footnote: By NeuroQOL convention, 5 = never, 4 = rarely, 3 = sometimes (2-3 times in the past 7 days), 2 = often (once a day), and 1 = very often (several times a day).

© 2024 Jaywant A et al. *JAMA Network Open.*

eFigure 2. Count of symptoms reported daily among those with a prior history of COVID-19

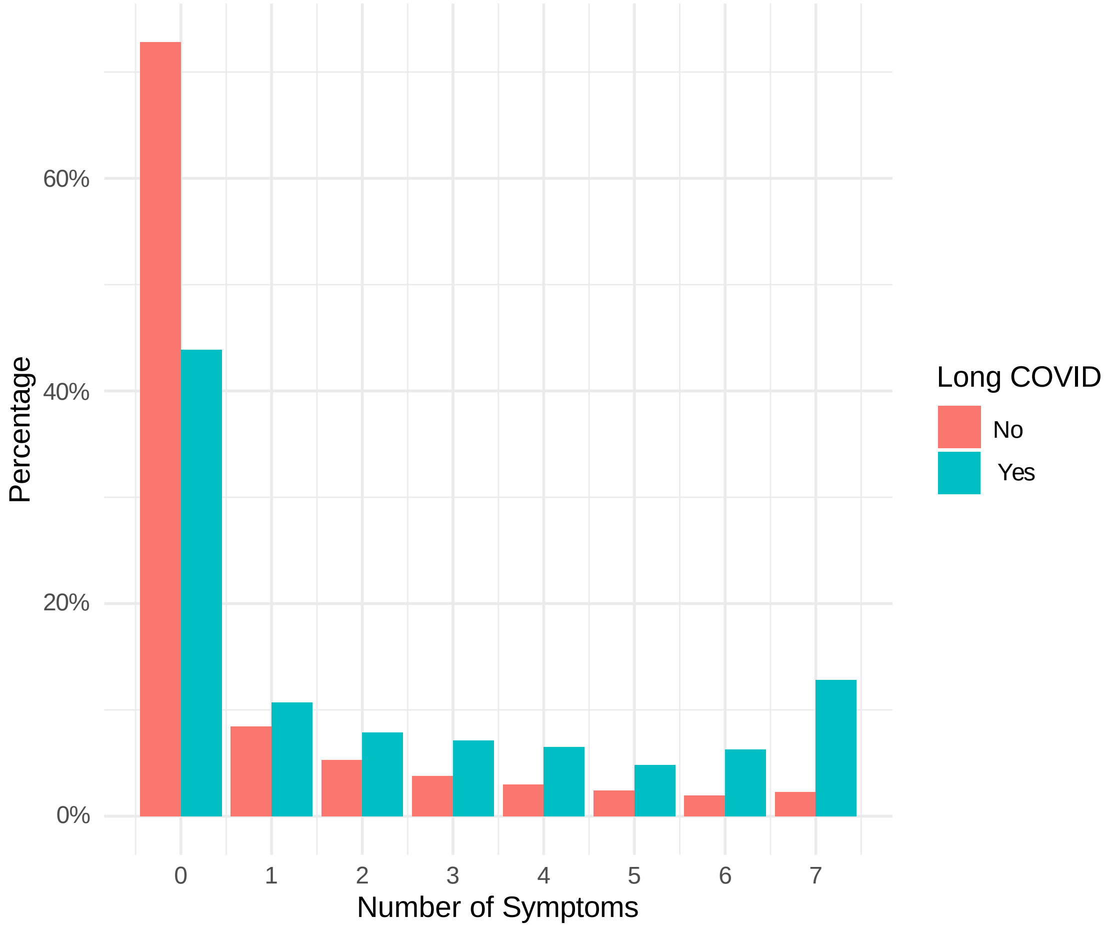

© 2024 Jaywant A et al. *JAMA Network Open.*

eTable. *Individuals with long COVID who did, or did not, report any cognitive symptoms occurring at least daily – row rather than column totals*

**Characteristic**	**No cognitive symptoms** N = 728[1]	**Cognitive symptoms** N = 955[1]
Age range		
18 to 24	27.4	72.6
25 to 34	35.8	64.2
35 to 44	42.4	57.6
45 to 54	37.5	62.5
55 to 64	46.9	53.1
65 and over	64.6	35.4
Gender		
Female	41.2	58.8
Male	51.1	48.9
Race and Ethnicity		
African American	46.5	53.5
Asian American	42.9	57.1
Hispanic	42.6	57.4
Other	47.4	52.6
White	42.8	57.2
Education		
Graduate Degree	43.2	56.8
College Degree	46	54
Some College	41.9	58.1
High School Graduate	41.9	58.1
Some High School or Less	37.9	62.1
Employment		
Full-time	44.2	55.8
Gig/Contract	10	90
Home-maker	37.8	62.2
Part-time	41	59
Retired	57.4	42.6
Self-employed	35.2	64.8
Student	32.6	67.4
Unemployed	32.7	67.3

© 2024 Jaywant A et al. *JAMA Network Open.*

Income		
<$25k	38.1	61.9
$25-<$50k	42.3	57.7
$50-<$100k	45.8	54.2
$100k+	46.5	53.5
Urbanicity		
Rural	41.3	58.7
Suburban	44.2	55.8
Urban	42.8	57.2

[1] %

© 2024 Jaywant A et al. *JAMA Network Open.*

eFigure 3. Linear regression model of PHQ-9

Variable		N	Estimate		p
**Age**	18 to 24	117		Reference	
	25 to 34	263		−0.34 (−1.59, 0.91)	0.594
	35 to 44	398		−1.38 (−2.56, −0.19)	0.023
	45 to 54	339		−1.75 (−2.96, −0.54)	0.005
	55 to 64	325		−3.01 (−4.23, −1.79)	<0.001
	65 and over	247		−4.58 (−5.88, −3.28)	<0.001
**Gender**	Female	1335		Reference	
	Male	354		0.33 (−0.36, 1.02)	0.354
**Education**	Graduate Degree	183		Reference	
	College Degree	563		−0.27 (−1.24, 0.70)	0.588
	Some College	524		0.50 (−0.50, 1.50)	0.327
	High School Graduate	362		0.96 (−0.12, 2.04)	0.080
	Some High School or Less	57		0.26 (−1.54, 2.05)	0.779
**Income**	<$25k	354		Reference	
	$25-<$50k	489		−0.61 (−1.41, 0.18)	0.132
	$50-<$100k	576		−1.39 (−2.20, −0.59)	<0.001
	$100k+	270		−2.48 (−3.47, −1.50)	<0.001
**Race and ethnicity**	African American	125		Reference	
	Asian American	28		−2.04 (−4.40, 0.32)	0.090
	Hispanic	147		0.10 (−1.27, 1.47)	0.885
	Other	78		−0.79 (−2.41, 0.84)	0.342
	White	1311		0.37 (−0.69, 1.44)	0.495
**as.factor(urban_type)**	Rural	400		Reference	
	Suburban	966		0.04 (−0.64, 0.72)	0.912
	Urban	323		0.07 (−0.79, 0.93)	0.879
**Number of daily cognitive symptoms**		1689		1.27 (1.17, 1.38)	<0.001

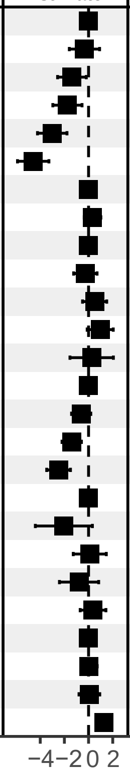

© 2024 Jaywant A et al. *JAMA Network Open.*

eFigure 4. Correlations between individual cognitive and PHQ-9 items

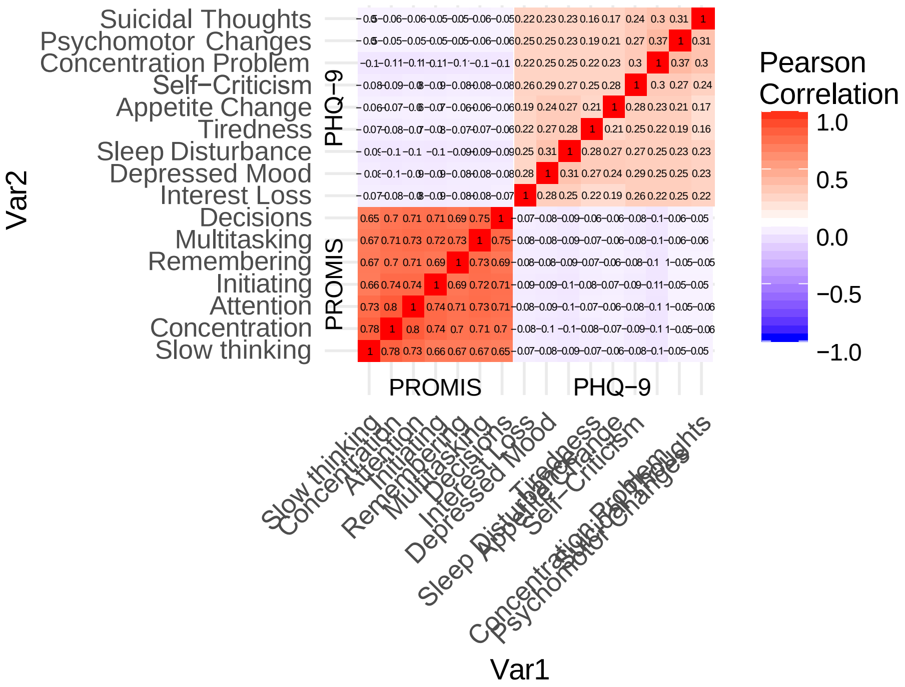

© 2024 Jaywant A et al. *JAMA Network Open.*

eFigure 5. Logistic regression model of full time employment, examining number of cognitive symptoms endorsed at least daily.

Variable		N	Odds ratio	p
**Age (years)**	18 to 24	117	Reference	
	25 to 34	271	1.87 (1.15, 3.06)	0.013
	35 to 44	394	1.55 (0.97, 2.47)	0.067
	45 to 54	344	0.97 (0.61, 1.57)	0.915
	55 to 64	319	0.51 (0.31, 0.83)	0.006
	65 and over	237	0.06 (0.03, 0.11)	<0.001
**Gender**	Female	1332	Reference	
	Male	350	1.72 (1.29, 2.30)	<0.001
**Education**	Graduate Degree	184	Reference	
	College Degree	557	0.51 (0.33, 0.76)	0.001
	Some College	525	0.34 (0.22, 0.52)	<0.001
	High School Graduate	358	0.31 (0.20, 0.49)	<0.001
	Some High School or Less	58	0.25 (0.11, 0.55)	<0.001
**Income**	<$25k	354	Reference	
	$25-<$50k	485	5.06 (3.45, 7.57)	<0.001
	$50-<$100k	572	6.82 (4.64, 10.21)	<0.001
	$100k+	271	8.15 (5.21, 12.96)	<0.001
**Race and Ethnicity**	African American	129	Reference	
	Asian American	28	0.97 (0.38, 2.57)	0.950
	Hispanic	155	1.17 (0.68, 1.99)	0.568
	Other	78	0.84 (0.44, 1.62)	0.609
	White	1292	0.96 (0.63, 1.48)	0.865
**Urbanicity**	Rural	397	Reference	
	Suburban	958	0.91 (0.68, 1.21)	0.506
	Urban	327	0.95 (0.66, 1.35)	0.759
**Number of daily cognitive symptoms**		1682	0.92 (0.88, 0.96)	<0.001

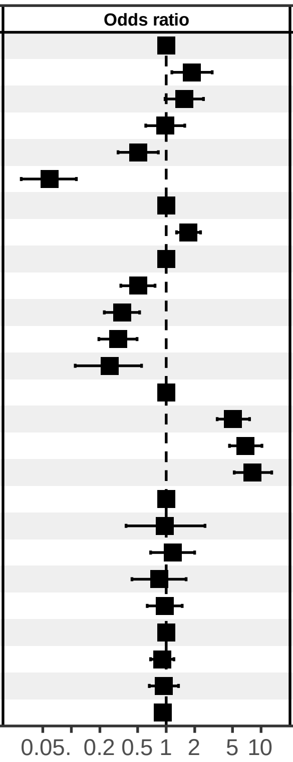

© 2024 Jaywant A et al. *JAMA Network Open.*

eFigure 6. Logistic regression model of moderate or greater functional impairment, examining number of cognitive symptoms endorsed at least daily, including PHQ9 total score.

Variable		N	Odds ratio	p
**Age (years)**	18 to 24	116	Reference	
	25 to 34	263	1.25 (0.78, 2.03)	0.36
	35 to 44	390	1.18 (0.75, 1.87)	0.48
	45 to 54	335	1.60 (1.01, 2.56)	0.05
	55 to 64	316	1.51 (0.94, 2.43)	0.09
	65 and over	233	1.55 (0.93, 2.61)	0.09
**Gender**	Female	1310	Reference	
	Male	343	0.90 (0.69, 1.19)	0.47
**Education**	Graduate Degree	181	Reference	
	College Degree	548	1.19 (0.81, 1.75)	0.38
	Some College	514	1.18 (0.80, 1.75)	0.41
	High School Graduate	353	1.42 (0.93, 2.17)	0.10
	Some High School or Less	57	1.14 (0.57, 2.26)	0.71
**Income**	<$25k	347	Reference	
	$25-<$50k	476	0.88 (0.65, 1.19)	0.41
	$50-<$100k	563	0.80 (0.59, 1.09)	0.15
	$100k+	267	0.93 (0.63, 1.36)	0.71
**Race and Ethnicity**	African American	124	Reference	
	Asian American	28	1.08 (0.43, 2.65)	0.86
	Hispanic	146	0.91 (0.54, 1.53)	0.72
	Other	77	0.73 (0.39, 1.37)	0.33
	White	1278	0.76 (0.51, 1.15)	0.19
**Urbanicity**	Rural	392	Reference	
	Suburban	942	0.72 (0.56, 0.94)	0.01
	Urban	319	0.81 (0.59, 1.13)	0.22
**Number of daily cognitive symptoms**		1653	1.27 (1.21, 1.33)	<0.001
**PHQ-9 total score**		1653	1.02 (1.00, 1.04)	0.05

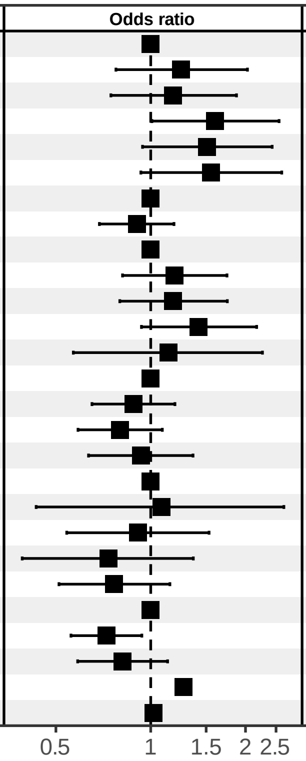

© 2024 Jaywant A et al. *JAMA Network Open.*

eFigure 7. Logistic regression model of full-time employment, examining number of cognitive symptoms endorsed at least daily, including PHQ9 total score.

Variable		N	Odds ratio	p
**Age (years)**	18 to 24	116	Reference	
	25 to 34	263	1.86 (1.14, 3.06)	0.014
	35 to 44	390	1.54 (0.96, 2.47)	0.072
	45 to 54	335	0.97 (0.61, 1.58)	0.916
	55 to 64	316	0.50 (0.30, 0.82)	0.006
	65 and over	233	0.05 (0.03, 0.11)	<0.001
**Gender**	Female	1310	Reference	
	Male	343	1.77 (1.32, 2.38)	<0.001
**Education**	Graduate Degree	181	Reference	
	College Degree	548	0.51 (0.33, 0.77)	0.002
	Some College	514	0.35 (0.23, 0.54)	<0.001
	High School Graduate	353	0.31 (0.19, 0.49)	<0.001
	Some High School or Less	57	0.26 (0.11, 0.56)	<0.001
**Income**	<$25k	347	Reference	
	$25-<$50k	476	4.83 (3.28, 7.24)	<0.001
	$50-<$100k	563	6.58 (4.47, 9.87)	<0.001
	$100k+	267	7.99 (5.09, 12.74)	<0.001
**Race and Ethnicity**	African American	124	Reference	
	Asian American	28	1.04 (0.40, 2.76)	0.939
	Hispanic	146	1.19 (0.69, 2.05)	0.543
	Other	77	0.87 (0.45, 1.69)	0.682
	White	1278	1.04 (0.67, 1.61)	0.860
**Urbanicity**	Rural	392	Reference	
	Suburban	942	0.89 (0.67, 1.19)	0.441
	Urban	319	0.90 (0.63, 1.29)	0.560
**Number of daily cognitive symptoms**		1653	0.92 (0.88, 0.97)	0.002
**PHQ-9 total score**		1653	1.00 (0.98, 1.02)	0.739

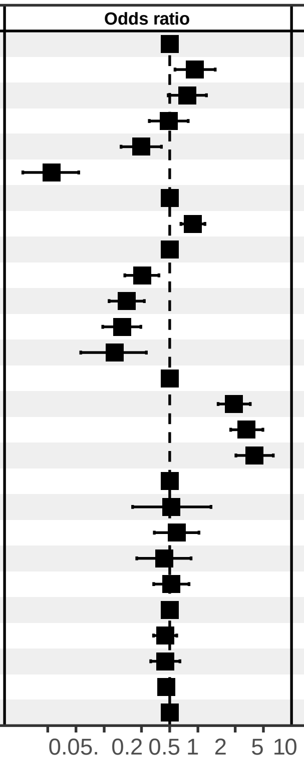

© 2024 Jaywant A et al. *JAMA Network Open.*
