# Supplementary material for: Cognitive Symptoms of Post–COVID-19 Condition and Daily Functioning
Source: JAMA Netw Open. 2024 Feb 14;7(2):e2356098. doi: 10.1001/jamanetworkopen.2023.56098 (PMC10867690; doi:10.1001/jamanetworkopen.2023.56098)
Supplement: Supplement 2. — Data Sharing Statement [file jamanetwopen-e2356098-s002.pdf]

Data Sharing Statement

Jaywant. Cognitive Symptoms of Post–COVID-19 Condition and Daily Functioning. *JAMA Netw Open*. Published February 14, 2024. doi:10.1001/jamanetworkopen.2023.56098

Data
Data available: Yes
Data types: Deidentified participant data
How to access data: Deidentified data will be made available to qualified investigators at the covidstates.org web site by June 2024.
When available: beginning date: 06-01-2024

Supporting Documents
Document types: None

Additional Information
Who can access the data: qualified investigators
Types of analyses: for a specified purpose
Mechanisms of data availability: after data access agreement
Any additional restrictions: none
